# Calcium Channel Expression and Applicability as Targeted Therapies in Melanoma

**DOI:** 10.1155/2015/587135

**Published:** 2015-02-01

**Authors:** A. Macià, J. Herreros, R. M. Martí, C. Cantí

**Affiliations:** ^1^University of Lleida-IRBLleida, 25198 Lleida, Spain; ^2^Department of Dermatology, University Hospital Arnau de Vilanova, 25198 Lleida, Spain

## Abstract

The remodeling of Ca^2+^ signaling is a common finding in cancer pathophysiology serving the purpose of facilitating proliferation, migration, or survival of cancer cells subjected to stressful conditions. One particular facet of these adaptive changes is the alteration of Ca^2+^ fluxes through the plasma membrane, as described in several studies. In this review, we summarize the current knowledge about the expression of different Ca^2+^ channels in the plasma membrane of melanoma cells and its impact on oncogenic Ca^2+^ signaling. In the last few years, new molecular components of Ca^2+^ influx pathways have been identified in melanoma cells. In addition, new links between Ca^2+^ homeostasis and specific cell processes important in melanoma tumor progression have been unveiled. Thus, not only do Ca^2+^ channels appear to have a potential as prognostic markers, but their pharmacological blockade or gene silencing is hinted as interesting therapeutic approaches.

## 1. Introduction

Ionized calcium (Ca^2+^) is a ubiquitous second messenger that mediates several physiological functions, such as cell proliferation, survival, apoptosis, migration, and gene expression. The concentration of Ca^2+^ in the extracellular milieu is 1-2 mM whereas, at rest, intracellular Ca^2+^ is maintained at about 100 nM [1]. Specific Ca^2+^-transporters and Ca^2+^-binding proteins are used by cells to extrude Ca^2+^ through the plasma membrane, transport Ca^2+^ into the intracellular reservoirs, and buffer cytosolic Ca^2+^ [2, 3]. Conversely, there is a diversity of Ca^2+^ channels in the plasma membrane allowing Ca^2+^ entry into the cytosol. Ca^2+^ influx may cross-talk with Ca^2+^ channels present in the endoplasmic reticulum (ER), resulting in localized Ca^2+^ elevations that are decoded through a variety of Ca^2+^-dependent effectors [1, 4].

It has been long known that external Ca^2+^ is needed to induce cell proliferation and cell cycle progression in mammalian cells [5]. Some studies indicate a requirement of Ca^2+^ influx to induce a G1/S-phase during the cell cycle process [6, 7]. However, in cancer cells such requirement is modulated by the degree of cellular transformation, so that neoplastic or transformed cells continue proliferating in Ca^2+^-deficient media [8].

Several types of Ca^2+^ channels have been involved in cell cycle progression: transient receptor potential melastatin (TRPM), transient receptor potential vanilloid (TRPV), Transient Receptor Potential Canonical (TRPC), components of the store-operated calcium entry (SOCE) pathway such as Ca^2+^ influx channel (ORAI1) and endoplasmic Ca^2+^ depletion sensor (STIM1), and voltage-gated calcium channels (VGCCs) [5]. Through the use of* in vitro* models, a role for TRPC1, ORAI1, or STIM1 in Ca^2+^ signaling changes associated with the proliferation of endothelial cells has been uncovered [9, 10]. In addition, L- and T-type VGCCs have been shown to be upregulated during the S-phase in vascular smooth muscle cells [11, 12]. T-type channels appear to be specially suited for promoting cell cycle progression by virtue of their fast activation upon weak depolarization. This feature enables transient elevations of cytosolic Ca^2+^ in nonexcitable cells that signal to favor mitotic progression through direct binding of Ca^2+^ to intracellular effectors such as calmodulin (CaM) [4].

Ca^2+^ influx also plays an important role in tumor growth. Commonly, cancer cells present alterations of Ca^2+^ fluxes across the plasma membrane that reflect changes in the expression, subcellular localization, and/or function of different types of Ca^2+^ channels [13, 14]. Among them, the expression of different members of the TRP family has been shown to be altered in cancer cells. Particularly, TRPC3 is induced in breast and ovarian epithelial tumors, and TRPC6 is highly expressed in cancer of breast, liver, stomach, and esophagus and glioblastoma [14]. Similarly, the expression of TRPV1 and TRV4 is elevated in human hepatoblastoma and breast cancer cells, respectively [14, 15], and the expression level of TRPV6 correlates with tumor progression in prostate, thyroid, colon, ovarian, and breast cancers [16]. Moreover, TRPM8 is overexpressed in different carcinomas and has been proposed to be a “prooncogenic receptor” in prostate cancer cells [16, 17].

In addition, depletion of Ca^2+^ from the ER may drive tumor growth by inducing Ca^2+^ influx through the plasma membrane, as the expression of the SOCE canonical components STIM1 and ORAI1 is augmented in various cancer types, including breast cancer, glioblastoma, melanoma, and esophageal carcinoma (reviewed in [1, 14]).

VGCCs are also involved in cancer progression by generating oscillatory Ca^2+^ waves that favor cell cycle progression [18]. Heightened levels of L-type channel Ca_v_1.2 mRNA have been reported in colorectal cancer [19]. Several studies have confirmed the increased expression of T-type Ca_v_3.2 channels in breast, colon, prostate, ovarian, esophageal, and colon cancers and in glioblastoma, hepatoma, and melanoma [20]. However, hypermethylation of the T-type channel gene CACNA1G (that encodes the Ca_v_3.1 isoform) occurs in different tumors including colon, pancreatic, and gastric cancer, suggesting that it acts as a tumor suppressor [21].

Cell physiology aspects other than proliferation are dependent on Ca^2+^ influx too. Through cell migration, Ca^2+^ signaling is involved in the directional sensing of the cells, in the redistribution and traction force of the cytoskeleton and in the repositioning of new focal adhesions [22, 23]. Cell migration is an early prerequisite for tumor metastasis with enormous impact on patient prognosis [23]. Members of the same Ca^2+^ channel families involved in tumor growth have been implicated in cancer cell migration and metastasis, such as TRP channels [24–26], STIM/ORAI-mediated SOCE [27–30], and T-type VGCCs [31, 32]. For example, TRPM7 has a promigratory effect on human nasopharyngeal carcinoma and its expression is related to metastasis formation [24], being a marker of poor prognosis in human breast cancer [25]. Nevertheless, TRPM1 expression in mice melanoma cells is reduced during metastasis [26]. Yang et al. provided evidence for the role of STIM1 and ORAI1 in the migration of the breast cancer cells using pharmacological blockers or siRNA [28]. The significance of STIM1 in focal adhesion and cell migration is extended to cervical cancer and hepatocellular carcinoma [29, 30]. Furthermore, it has been shown that T-type calcium channels regulate cell motility and migration in fibrosarcoma cells [31]. Conversely, Zhang et al. provided evidence for T-type channel blockers as dual inhibitors of proliferation and migration of human glioblastoma cells [32].

Finally, cell fate is also dependent on Ca^2+^ influx and its molecular machinery. Both the pharmacological blockade and the siRNA-mediated silencing of TRPM8 channels have been shown to induce the apoptotic death of prostate cancer cells [33], indicating a critical role for these channels in Ca^2+^ homeostasis maintenance. It has been suggested that TRPM8 could regulate either proliferation or apoptosis mechanism in prostate cells, depending on its intracellular localization [34]. Moreover, TRPV1 has been proposed as a useful target for killing malignant cells, since mitochondrial function was inhibited and apoptosis was induced in pancreatic cancer cells treated with a vanilloid analogue [8, 35]. VGCCs also play a relevant role in the survival of cancer cells. We have recently reported that T-type pharmacological blockers induce apoptosis in melanoma cells, in addition to reducing its proliferation [36]. Importantly, in the referred work the pharmacological results were backed up by siRNA-mediated silencing of Ca_v_3.1 and Ca_v_3.2 T-type channel isoforms. Likewise, Valerie et al. found that inhibition of T-type channels by a selective antagonist or siRNA-mediated gene knockdown not only reduced glioma cell viability but also induced apoptosis. These effects were reached via inhibition of the mTORC2/Akt pathway followed by a reduction in the phosphorylation of antiapoptotic Bad [37].

Hereon, this review will discuss the current knowledge about the role of different Ca^2+^ channels expressed in the plasma membrane of melanoma cells, as well as the Ca^2+^ signaling pathways involved during tumorigenesis and tumor progression.

## 2. Calcium Channels in Melanoma

Cutaneous melanoma is a malignant skin cancer that arises from transformed melanocytes* de novo* or from dysplastic, congenital, or common nevi [50]. Melanoma is the most dangerous form of skin cancer, and its incidence is steadily increasing worldwide. In spite of being the subject of intense laboratory investigations and numerous clinical trials, the prognosis of metastatic melanoma is still poor. New treatment strategies such as immunotherapy and specific gene therapy are currently under investigation.

### 2.1. Transient Receptor Potential Melastatin (TRPM) in Melanoma

TRP channels are known to regulate melanocyte physiology, particularly members of the TRPM subfamily [38]. Untransformed melanocytes express the full-length TRPM1 mRNA along with an alternative splicing variant (TRPM1-s) [51]. TRPM1 function appears to be critical to normal melanocyte pigmentation and melanogenesis, and thus this channel is a potential target for pigmentation disorders [52].

**Figure 1:**
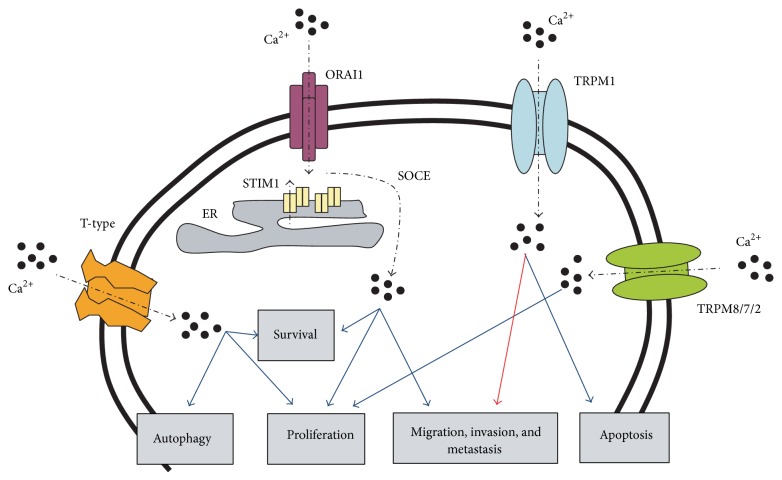
Ca^2+^-influx pathways and their physiological functions in melanoma cells. Blue line indicates positive regulation. Red line indicates inhibition.

TRPM1 was first discovered in B16 mouse melanoma cell lines as a result of a differential display analysis [26]. This channel is strongly expressed in poorly metastatic B16 cells and expressed at reduced levels in the highly metastatic B16-F10 variant [26]. Moreover, in formalin-fixed tissue sections benign nevi were found to express high levels of TRPM1 that showed a low expression in primary melanomas whereas the full-length transcripts were not detected in melanoma metastases (but several short fragments of TRPM1) [26, 39]. As a matter of fact, several studies point to TRPM1 as a tumor suppressor in melanoma cells, as its loss of expression correlates with melanocytic tumor progression, metastatic potential, tumor thickness, and overall melanoma tumor aggressiveness (Figure 1; Table 1) [16, 26, 38–41]. In line with this, it has been suggested that the levels of TRPM1 mRNA can be used to predict the future development of metastatic melanoma [16, 38].

The regulation of TRPM1 gene expression has been extensively investigated. It has been proposed that TRPM1 expression in melanocytes and melanoma cells is regulated by a promoter region of the gene that contains four microphthalmia transcription factor (MITF) binding sites. Several groups demonstrated that MITF directly regulates the expression of TRPM1* in vitro* and* in vivo* during melanoma progression [38, 42, 53, 54].

TRPM1 gene encodes both TRPM1 mRNA and miR-211 which is coded by the sixth intron of the gene. TRPM1 and miR-211 share the same promoter and are coregulated by MITF. Similar to TRPM1 protein, miR-211 is highly expressed in melanocytes and nevi and is reduced in melanoma cells [55, 56]. Consistently, overexpression of miR-211 exhibited significant growth inhibition and reduced migration and invasion in melanoma cells [38, 55–57].

Melanoma cells also express functional TRPM8 channels that produce a sustainable Ca^2+^ influx upon activation by menthol as agonist [43]. Strikingly, in this study the viability of melanoma cells was dose-dependently depressed in the presence of menthol, indicating that these channels underlie tumor progression via the Ca^2+^ handling pathway and suggesting TRPM8 Ca^2+^ channels as novel targets of drug development for malignant melanoma (Figure 1; Table 1).

Another member of the TRP family, TRPM2, is an ion channel capable of conferring susceptibility to cell death upon oxidative stress [58]. Quantitative RT-PCR experiments revealed that two antisense transcripts (TRPM2-AS and TRPM2-TE) from the TRPM2 gene were upregulated in melanoma cells and that their activation was linked to the hypermethylation of a shared CpG island. Moreover, knockdown of TRPM2-TE (proposed as a dominant-negative transcript) increased the vulnerability of melanoma cells to undergo apoptosis and necrosis, and overexpression of wild-type TRPM2 in melanoma cells leads to a faster proliferation (Figure 1; Table 1) [38, 44].

Finally, TRPM7 receptor has a protective and detoxifying function in normal and malignant melanocytes. In contrast to TRPM1, TRPM7 is highly expressed in metastatic melanoma (Figure 1; Table 1) [38, 45].

### 2.2. Store-Operated Ca^2+^ Entry (SOCE) in Melanoma

Ca^2+^ storage in the ER is an essential indicator of the proliferative, metabolic, and apoptotic status of cells. The retrograde signaling process from ER Ca^2+^ depletion to SOCE activation has a central role in many cellular and physiological functions. Indeed, SOCE is the main mechanism that implicates Ca^2+^ import from extracellular to intracellular space, especially in nonexcitable cells [59]. In the SOCE pathway, STIM proteins detect the depletion of Ca^2+^ at the ER and respond by translocating to the plasma membrane, where they activate ORAI Ca^2+^ channels and allow Ca^2+^ influx.

However, the role of SOCE in melanoma has not been investigated extensively. One study suggested a functional relevance for Ca^2+^-driven growth and survival of melanoma cells due to the control of SOCE by mitochondria [46]. The authors showed that coupling of mitochondria to SOCE allows the maintenance of strong Ca^2+^ fluxes and sustains a constitutive activation of PKB/Akt pathway, leading to an increased melanoma cell survival and resistance to apoptosis (Figure 1; Table 1). The same research group later demonstrated* in vitro* and* in vivo* that lipid rafts are critical for coupling SOCE to the constitutive activation of PKB/Akt in a Ca^2+^/calmodulin-, Src-, and PP2A-mediated pathway. These results underscore the potential of lipid raft disruptors as effective anticancer treatments [47].

More recently, Umemura et al. described that proliferation and migration are also regulated by SOCE in melanoma cells. They found that STIM1 and ORAI1 were expressed at high levels in human melanomas and melanoma cell lines. When SOCE activity was inhibited by pharmacological blockade or by siRNA-mediated gene knockdown, the proliferation rate was halted, cell migration was prevented, and metastasis progression was delayed (Figure 1; Table 1). Moreover, their results suggested that melanoma progression is promoted by SOCE through CaMKII/Raf-1/ERK signaling pathway, independently of BRAF mutations. Therefore, targeting SOCE could be a new strategy to treat a great number of melanoma patients, in monotherapy or in combination with BRAF inhibitors [48].

### 2.3. Voltage-Gated Ca^2+^ Channels (VGCCs) in Melanoma

In 2012, our research group reported that both normal melanocytes and transformed melanoma cells express functional VGCCs, including members of the Ca_v_1 (L-type), Ca_v_2 (N, P/Q or R-types), and Ca_v_3 (T-type) families [49]. However, differences were noticed between the different cell lines regarding the expression of particular isoforms. Remarkably, untransformed melanocytes expressed only very low levels of T-type Ca_v_3.1 channels, whereas transcripts for T-type Ca_v_3.2 and Ca_v_3.3 channels were undetectable. We also found a correlation between the proliferation rate and the expression of specific T-type channels isoforms, such that the melanoma cell lines displaying a high proliferation rate expressed higher levels of Ca_v_3.2 channels (Figure 1; Table 1), whereas those ones growing slowly expressed preferentially the Ca_v_3.1 isoform. Interestingly, the expression of these two T-type channel isoforms was counterbalanced. Furthermore, the results attained in gene knockdown experiments showed that whereas both Ca_v_3.1 and Ca_v_3.2 isoforms promote the progression of melanoma cells, the expression of Ca_v_3.1 is associated with slow cycling and it is induced under hypoxic conditions [49]. In a follow-up study, we described that clinically used T-type channel pharmacological blockers induced G1/S cell cycle arrest and also triggered the apoptotic death of melanoma cells, which was partially dependent on mitochondrial caspase activation [36]. An in-depth analysis of the process revealed that apoptosis is preceded by ER stress and subsequent inhibition of the autophagic flux, which we found to be constitutively activated in melanoma cells (Figure 1; Table 1). These effects were mimicked by knockdown of Ca_v_3.1 and Ca_v_3.2 channels, thus allowing the identification of these T-type channels as novel targets to deregulate autophagy and induce cytotoxicity in melanoma cells [36].

## 3. Conclusions and Future Developments

Recent advances in the understanding of how Ca^2+^ influx is involved in melanoma tumorigenesis and progression may provide new targeted therapies for melanoma treatment. For this to materialize, it is essential to further deepen the study of the role of plasma membrane Ca^2+^ channels in melanoma cell proliferation and also in other less explored areas, such as migration or survival. In light of current knowledge, the selective activation of certain TRP channel isoforms and/or the development of humanized inhibitory antibodies to extracellular domains of TRP channels arise as putative therapeutic strategies [15, 16, 51]. Also, the blockade of Ca^2+^ influx through the use of pharmacological SOCE blockers and/or raft-targeting agents appears as an interesting approach to tackle melanoma progression [47, 48]. Finally, we highlight the potential of T-type Ca^2+^ channels as novel prognosis markers and/or therapeutic targets in melanoma. In this regard, our studies have uncovered a dual role of T-type Ca^2+^ channels in controlling melanoma cell proliferation and Ca^2+^ homeostasis, with an impact on adaptive cancer cell mechanisms like ER stress and macroautophagy that are often associated with chemotherapeutic or radiotherapeutic resistance [36, 49]. Thus, existing Ca^2+^ channel blockers may expand the pharmacological arsenal and become valuable partners for combined chemotherapies against melanoma.

## Figures and Tables

**Table 1:** Expression and physiological role of calcium channels in melanoma.

Ca^2+^ channel	Expression in melanoma cells relative to melanocytes	Effects	References
TRPM1	Very low/undetectable	Proapoptotic Reduced metastatic potential Reduced migration and invasion	[16, 26, 38–42]
TPRM8	Increased	Favored tumor progression	[17, 43]
TRPM2	Increased	Increased proliferation by overexpression	[38, 44]
TRPM7	Increased	Favored metastasis and invasion	[38, 45]
SOCE	Increased	Prosurvival Increased proliferation	[46–48]
T-type	Increased	Increased proliferation Macroautophagy blocked by pharmacological inhibition/gene silencing	[36, 49]

## Abbreviations

ER: Endoplasmic reticulum
Ca^2+^: Ionized calcium
TRPM: Transient receptor potential melastatin
TRPV: Transient receptor potential vanilloid
TRPC: Transient receptor potential canonical
SOCE: Store-operated calcium entry
VGCCs: Voltage-gated calcium channels
BRAF: v-raf murine sarcoma viral oncogene homolog B1.
